# A correlation study of emergency department nurses’ fatigue, perceived stress, social support and self-efficacy in grade III A hospitals of Xi’an

**DOI:** 10.1097/MD.0000000000021052

**Published:** 2020-08-07

**Authors:** Chao Wu, Yiling Ge, Chao Xu, Xinyan Zhang, Hongjuan Lang

**Affiliations:** a Air Force Medical university, Xi’an; b Army 75 group military hospital, Kungming, China.

**Keywords:** emergence department, fatigue, nurses, perceived stress, self-efficacy, social support

## Abstract

Fatigue is a universal and challenging problem in a nurse's career, particularly for those working in the emergency department. Through analyzing the current status of emergency department nurses’ fatigue, the purpose of this study is to provide guidance for occupational health promotion strategies making and fatigue relief.

Cross-sectional study was conducted among nurses working in emergency department in 6 grade III A hospitals in Xi’an, China. Convenience sample of 346 nurses agreed to participate in this study. Data collection was based on the questionnaires. Descriptive statistics, hypothesis tests and correlation analysis were used to describe the samples’ characteristics and identify associations amongst participants’ characteristics.

The fatigue score of those emergency nurses from grade III A hospitals in Xian was 8.71 ± 3.01, a high fatigue level. Moreover, there were significant differences in fatigue scores of different age groups, sleep qualities, work stress levels and physical states (*P* < .01). Further, the dimension of physical fatigue in various age groups, job title, marital status, sleep qualities, work stress levels and physical states was significantly different (*P* < .05) and the dimension of mental fatigue with different sleep qualities, work stress levels and physical states was significantly different (*P* < .01). The results of correlation analysis showed that fatigue was positively correlated with perceived stress while negatively correlated with social support and self-efficacy (*P* < .01). The multiple stepwise linear regression analysis indicated that the independent variables in the fatigue regression equation were perceived stress, physical condition and work stress in turn (*P* < .01), and the independent variables in the the dimensions of physical fatigue regression equation were perceived stress, physical condition,work stress and job title in turn (*P* < .05); the independent variables in the the dimensions of mental fatigue regression equation were perceived stress,subjective support and physical condition in turn (*P* < .05).

The current status of high fatigue level of emergency nurses should be taken seriously. It is imperative to take effective measures to help emergency nurses reduce stress, improve social support, promote the self-efficacy, and thus relieve fatigue.

## 1 Introduction

Fatigue is a common and serious problem in all walks of life, especially in the highly stressful and loaded medical profession. Diagnostic Interview Schedule reported that fatigue unexplained by a medical etiology has lifetime prevalence of 20% to 25%.^[1]^ Fatigue can cause job burnout, low work efficiency, and even lead to serious medical disputes and medical accidents.^[2]^ In terms of nursing service, it has been stated that fatigue of nurses could cause professional burnout sense and lower productivity.^[3]^ Given the tremendous risks of fatigue, there is a widespread concern about fatigue among medical staff.

Fatigue is a subjective sense of discomfort, known as the first leading cause of sub-health. In the clinical work, fatigue is defined as a sustaining and intense feeling of burnout and powerlessness when engaged in physical or mental activities, and it can not be relieved in the short term.^[4]^ As a common complaint, high levels of fatigue were associated with a great burden of psychiatric symptoms, poor cognitive and physical functioning. Of note, it was reported that moderate/sever fatigue were related to greater suicidality, which implicated the possible link between fatigue and suicidal behavior.^[1]^ Perceived stress refers to the confusion and threat caused by various stimulants and negative events in life.^[5]^ Social support is not merely an objective support which is a visibly material assistance and participation of social network. Social support can also be a subjective and emotional support, which is an emotional experience and satisfaction when an individual is respected, supported and understood in the society.^[6]^ Self-efficacy refers to the individual's evaluation and judgment on whether they are capable of accomplishing something, which reflects the degree of individual self-confidence.^[7]^

Known as the underdeveloped part of China, the northwest area of China is also lack of medical resources.^[8]^ Xi’an is the capital of Shanxi province and also an important city in northwest China, playing a pivotal role in politics, economy, education and medical treatment. Therefore, the grade III A hospitals in Xi’an have wide radiation range within northwest China and undertake heavy home visit tasks from all around. Consequently, working in the emergency departments of those hospitals, nurses have to deal with a large number of patients, especially many critically ill patients in danger with rapid progression.^[9,10]^ Accordingly, those nurses work under great strain and more prone to fatigue. Therefore, it is instructive to explore the fatigue status of the emergency nurses.

According to the definition in Classification of Diseases Manual, job burnout is caused by chronic stress and has not been managed effectively.^[11]^ Several prospective and high-quality studies have shown the severe consequences of job burnout for the workers’ physical, psychological and occupational well-being and the associations between job burnout and many health problems.^[12]^ According to the results of numerous previous researches, job burnout was significantly related to and interacted with perceived stress while negatively correlated with social support.^[13]^ And job burnout can easily lead to fatigue.^[14]^ Interestingly, studies also found that social support could relieve the work pressure and fatigue^[15]^ and Molero^[16]^ found that self-efficacy can predict perceived stress in nursing professionals through a cross-sectional study, which implies the possible correlation among fatigue, perceived stress, social support and self-efficacy.

Despite the researches of nurses’ fatigue, burnout and mental health have been topics of great interest,^[17,18]^ in China, there is no relevant researches on the association among nurses’ fatigue, stress perception,social support and self-efficacy in the emergency ward of grade III A hospitals yet. Consequently, it is essential and very significant to carry out this investigation to address the gap in the research literature.

The purpose of this study is to explore the current status and its influence factors of emergency ward nurses’ fatigue, and further provide theoretical basis for the health promotion policies making, to relieve fatigue and improve work efficiency in nursing practice.

## 2 3.Methods

### 2.1 3.1.Design

A cross-sectional study with data collection on the basis of questionnaires.

### 2.2 Setting

This study was conducted in the emergency wards of 6 grade III A hospitals in Xi’an, includeing Xijing hospital-the first affiliated hospital of Air Force Medical University, Tangdu hospital-the second affiliated hospital of Air Force Medical University and the second affiliated hospital of Xi’an Jiaotong University, and so on.

### 2.3 Participants

Convenience sample of 346 nurses (response rate,93.01%)agreed to participate in the study, with those who are on leave or pregnancy and those who have recently suffered major life events being excluded. The questionnaire was issued on the spot and was filled out within 30 minutes. The investigators sorted out and verified the recovered questionnaires on the spot, and corrected the missing or wrong questionnaires in time to ensure the quality of questionnaires recovery. Finally, a total of 350 questionnaires were distributed and 346 valid questionnaires were collected. Of the 4 invalid questionnaires, 3 were excluded due to incomplete information response (respondents had not completed all the questions in the questionnaires) and the other 1 was due to non-standard information response (the respondent had chosen 2 options in response to an 1-choice question).

### 2.4 Procedure

The data were collected between March and May 2019 under the instruction of trained investigators.

### 2.5 Data collection tool

Questionnaire was applied to conduct the survey, which is consisted of 5 sections. The first part was the general information questionnaire, including ages, work units, positions, titles, education backgrounds, marital status and salary, etc. The second part was the fatigue scale, composed of 14 items about physical fatigue and mental fatigue.^[19]^ The third part was Chinese Perceived Stress Scales, containing 14 items and including 2 dimensions of feelings of tension and losing control.^[20]^ In the forth part, Social Support Rating Scale, 3 dimensions of objective support, subjective support and the utilization of social support were evaluated with 10 items.^[21]^ The last part was General Self-Efficacy Scale.^[16]^ All scales have passed the test of reliability and validity, and have good reliability and validity.

### 2.6 Ethical considerations

This study was conducted according to the ethical guidelines described in the Helsinki Declaration (World Medical Association, 2013). And informed consent was obtained from the participants.

## 3 Data analysis

Descriptive statistics (mean, standard deviation, frequency and percentage) were used to describe the sample's characteristics. T-test and variance analysis were applied for univariate analysis, and pearson correlation analysis and multiple linear regression analysis were applied for multivariate analysis. The normal distribution and types of variables were considered to decide which tests were suitable for the analysis. All data were input in Excel and analyzed with SPSS Statistics for Windows, version 20.0. Significance was assessed at *P* < .05 level in the bivariate analysis.

## 4 Results

Participants’ profile: The majority of the participants were between 20 and 40 years (n = 323; 93.36%), 71.10% were married (n = 246), 76.88% had a bachelor's degree (n = 266). Nurses accounted for 27.17% (n = 94), senior nurse 57.51% (n = 199). 51.73% were exposed in high work stress (n = 17) and 69.36% (n = 24) were in sub-health. (Table 1).

**Table 1:**
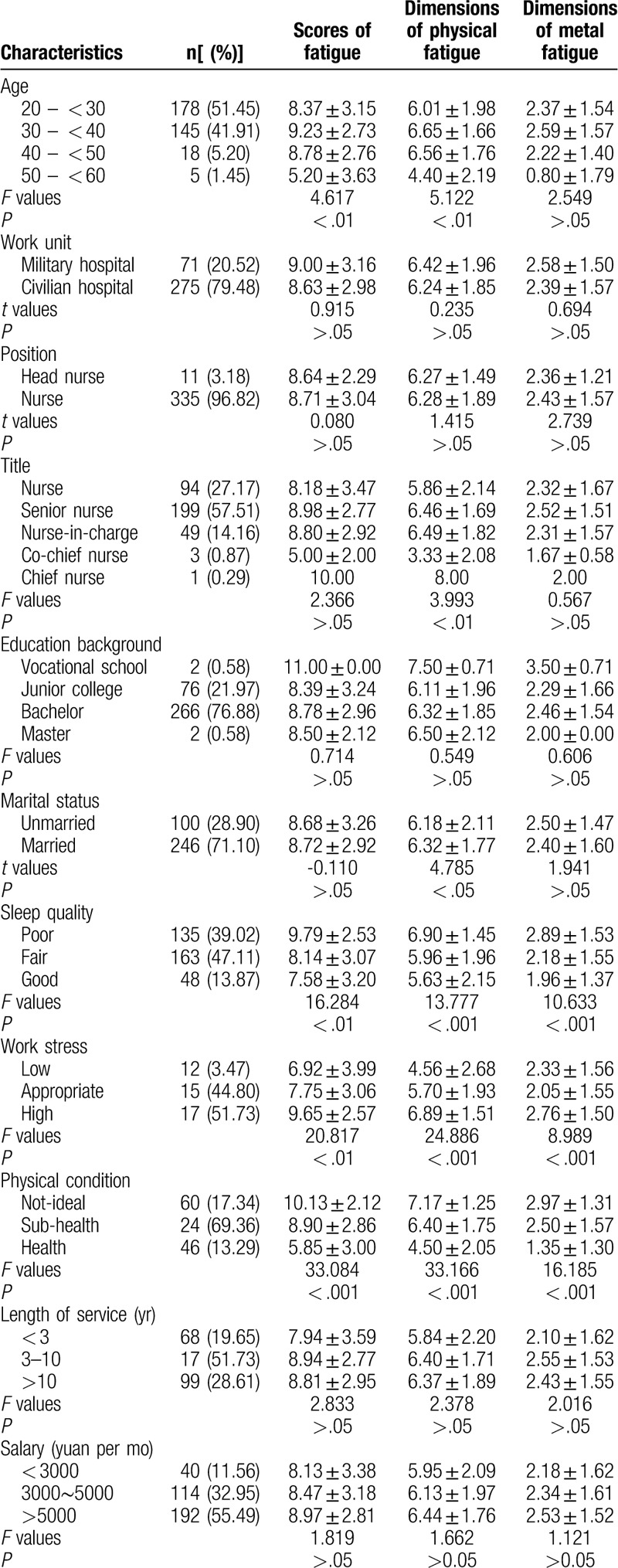
The univariate analysis of general information and fatigue (n = 346, ± s).

The univariate analysis between general information and fatigue indicated that there were significant differences in fatigue scores of different ages, sleep qualities, working stress and physical conditions (*P* < .01). And in terms of the 2 dimensions of fatigue, different age groups, job title, marital status, sleep qualities, work stress levels and physical states were significantly different in physical fatigue (*P* < .05) and different sleep qualities, work stress levels and physical states were significantly different in mental fatigue (*P* < .01) (Table 1).

The correlation analysis of fatigue, perceived stress, social support and self-efficacy indicated that fatigue was positively correlated with perceived stress (*r* = 0.491, *P* < .01) and negatively correlated with social support (*r* = −0.271, *P* < .01)and self-efficacy (r = −0.358, *P* < .01)(Table 2).

**Table 2:**
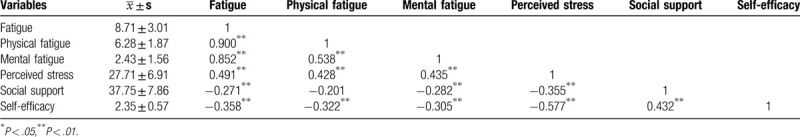
Correlation analysis of nurses’ fatigue with perceived stress and social support.

Multiple linear regression analysis in influence factors of nurses’ fatigue in emergency department of grade III A hospitals in Xi ’an was conducted with total fatigue score, including 2 dimensions of physical fatigue and mental fatigue, as the dependent variables. We assign values to independent variables (Table 3). The independent variables were stress perception, social support, self-efficacy and the statistically significant variables of univariate analysis (entry level was 0.05, deletion level was 0.10). According to the results of the regression equations significance, the independent variables in the fatigue regression equation were perceived stress, physical condition and work stress in turn (Modle1: F = 51.724, *P* < .01), and the independent variables in the dimension of physical fatigue regression equation were perceived stress, physical condition,work stress and job title in turn (Modle2: *F* = 44.179, *P* < .05); the independent variables in the dimension of mental fatigue regression equation were perceived stress,subjective support and physical condition in turn (Modle3: *F* = 33.093, *P* < .05) (Table 4).

**Table 3:**
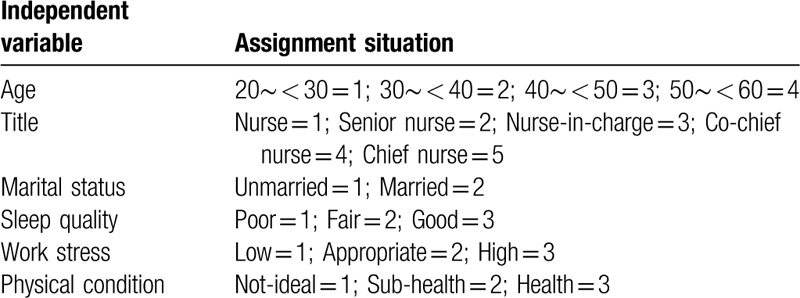
Assignment of independent variable.

**Table 4:**
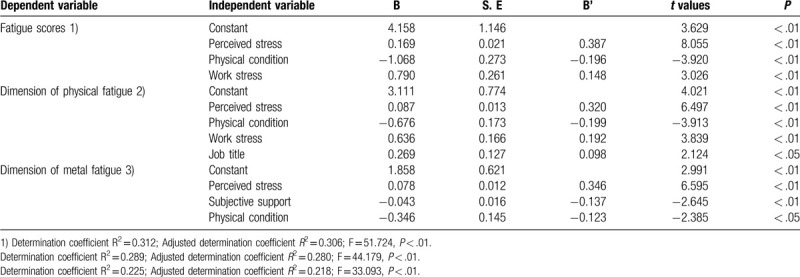
Multiple linear regression analysis of factors influencing nurses fatigue in emergency department.

## 5 Discussion

### 5.1 The current fatigue situation of nurses in Xi’an

As demonstrated by the findings, the overall fatigue score of nurses working in the emergency department of the grade III A hospitals in xi ’an was 8.71 ± 3.01, body fatigue was 6.28 ± 1.87 and mental fatigue was 2.43 ± 1.56. This survey revealed that the emergency nurses were in a high fatigue level, which was consistent with the result of a relevant survey conducted in the United States.^[22]^ Long working length and heavy workload are easy to produce job burnout^[23]^ and lead to fatigue. As can be seen from the scores of the 2 dimensions of fatigue, the fatigue of those nurses mainly came from physical fatigue. This may be related to nurses’ mechanical and repetitive work operations, as well as physical night shift pattern. Particularly, in comparison with other hospital departments, emergency work is highly riskier and overloaded. Consequently, tremendous pressure, mechanical repetitions, disrupted body clocks and stressful relationships, these unique features of working as an emergency nurse, probably are responsible for the high level of fatigue for those nurses.^[15]^ In the clinical work, we should not only pay attention to the diseases and their therapies, but also attach importance to the subjects who implement the medical practice, especially in terms of psychology, which is directly related to the quality of medical service.

### 5.2 Analysis of the influencing factors of nurses’ fatigue in emergency department

#### 5.2.1 Perceived stress

As listed in Table 2, there was a positive correlation between fatigue and stress perception (*P* < .01). And the multiple linear regression analysis showed that stress perception is the primary influencing factor of fatigue scores, in the 2 dimensions of physical and mental fatigue (Table 4). As the primary factor affecting fatigue of emergency nurses, perceived stress refers to the perceptible disturbance or threat caused by various stimulus factors and negative events in life, and result in physical and psychological stress and discomfort. It was reported that the greater the perceived stress, the more fatigued the individual is.^[18]^ The emergency departments of grade III A hospitals admit a large number of critical patients, especially those with complex and unstable conditions. So there is no wonder that the emergency nurses of grade III A hospitals are exposed in the heavy occupational load. Besides, the emergency nurses’ work is also challenging and demanding. For example, there are various kinds of sophisticated rescue equipment requiring skilled mastering. Hence the heavy workload and high demands of emergency work expose nurses in the state of high perceived stress, and the fatigue increases accordingly.^[24]^ To reduce perceived stress and improve professional satisfaction, nursers should pay attention to work experience accumulation and operational proficiency. Interestingly, a study in Ireland found that moderate alcohol intake could reduce stress on the job for emergency nurses,^[6]^ hence having a moderate drinking after work may be another good way for those stressful nurses. From the managers’ perspective, it is advisable to develop reward mechanism to motivate workers and adopt flexible scheduling.^[25]^ Furthermore, resiliency should be included as an important assessment index for the recruitment and professional evaluation of emergency nurses.^[26]^

#### 5.2.2 Social support

As listed in Table 2, there was a negative correlation between fatigue and social support (*P* < .01). In Table 4, subjective support enters the regression equation of mental fatigue, which means subjective support is the influencing factor of mental fatigue and both of them belong to subjective category. Social support consists of objective support, subjective support and utilization of support. Social support can play a soothing role to a certain extent, particularly the spiritual support can give a great motivation for individuals to face and handle fatigue problem. Huang^[27]^ asserted that providing more support was a crucial strategy to reduce fatigue among nurses. Combined with the result of our study, providing subjective support may contribute to the fatigue relief. Consequently, the managers should pay attention to the construction of harmonious circumstance in department and provide regular psychological counseling, especially for those who experienced bereavement.^[28]^ The nurses’ family should also build cozy domestic environment and harmonious family atmosphere.^[29]^

#### 5.2.3 Self-efficacy

The self-efficacy score of nurses in the emergency department of Xi’an was (2.35 ± 0.57), which was at a low level. And as listed in Table 2, fatigue was negatively correlated with self-efficacy (*P* < .01). Similarly, Breukers^[30]^ found that self-efficacy can reduce the fatigue of individuals. Self-efficacy reflects the level of self-confidence and a good sense of self-efficacy can also enhance the self satisfaction, which could help nurses develop a deep interest and commitment in their occupation, reduce the perceived working stress, drive them handle stress positively and recover from setbacks quickly and thus relieve fatigue in nursing professionals. So self-efficacy is an important psychological trait for nurses. Managers could popularize the skills of building self-efficacy by lectures and nurses themselves also should pay attention to fostering this psychological competence.

#### 5.2.4 Physical condition

The multiple linear regression analysis of fatigue score illustrated that physical condition was the influencing factor of fatigue, and both in the 2 dimensions of physical and mental fatigue (*P* < .01) (Table 4), which was consistent with the result of a previous study that reported people in good physical condition are in less fatigue.^[31]^ The nurses in emergency department do not merely handle high-intensity work during the daytime, but also work in long-term night shift mode, which would lead to the disturbance of the circadian rhythm of the human body, and result in insomnia, fatigue and other symptoms.^[32]^ In addition, some research reported that the prevalence rate of various diseases, such as hypertension, anxiety and depression, nurses in emergency department was higher than the nurses in the ward.^[33]^ A New Zealand study of nurses also found that nurses were more likely to have chronic sleep problems and the long-term night shift pattern could lead to sleep disorders.^[34]^ Therefore, long-term shift work, irregular daily schedule and high load of work pressure expose the nurses of the emergency department to overdraft condition, causing high fatigue.^[35]^ The managers should enhance scientific duty shift, reduce the frequency of night shift scheduling and improve the shift and off duty system. Besides, it is imperative to organize regular physical examination for nursing staff to detect and prevent diseases timely. Notably, in consideration of the possible link between fatigue and suicidal risks, some specific biological factors which may be associated with and even predict a suicide attempt, such as prolactin and thyroid hormones, could be detected regularly,^[36]^ especially for those who have experienced bereavement.^[28]^ A healthy lifestyle can reduce anxiety and fatigue.^[37,38]^ So for those nurses, having a healthy lifestyle like balanced diet, regular exercise, and enjoying recreation properly may help maintain physical fitness and relieve fatigue.^[39,40]^

#### 5.2.5 Work stress

In Table 4, work stress enters the regression equation of the total fatigue score and the dimension of physical fatigue (*P* < .01), which was consistent with the results of previous researches.^[41,42]^ Work stress is different from pressure perception, which is objective and comes from external pressure. Work pressure refers to the imbalance between the requirements of work and people's capacity. It is not merely a powerful driving force, but could become a negative factor damaging work performance and occupational health. Researches showed that emergency department nurses met with a high risk of occupational exposure.^[43]^ Dealing with critically ill patients and frequent night shift expose nurses to a state of high tension for a long time.^[44]^ Moreover, nurses also have to deal with complex interpersonal relationship with consultation doctors from various departments and the anxious patients family. Those all could generate work pressure. On the other hand, high levels of fatigue can further lead to a vicious circle of inefficiency and stress over errors.^[45]^ In view of this situation, to relieve the fatigue caused by work stress, managers could organize regular psychological counseling and job training, arrange rational shift scheduling and the emergency nurses themselves should take initiative to increase resilience and self-adjust, such as practice more to improve operational proficiency, enjoy recreation properly.

## 6 Strengths and limitations

Currently, there is still no study of emergency nurses regarding the relationship between fatigue and stress perception social support and self-efficacy in China. This paper has discussed the relationship between the 4 elements, developing guidance for fatigue alleviation. However, there are still some limitations in this paper. Initially, the investigation only was launched in the grade III A hospitals of xi ’an, with limitations of respondents and regions. If accessible, it is more meaningful to implement cross-sectional analysis in multiple cities or even countries. Besides, this paper just explored the 4 factors of fatigue, stress perception self-efficact and social support. In the future, we can try to investigate nurses from more aspects.

## 7 Conclusion

Our study found that the fatigue of emergency nurses is at a high level. And the fatigue is correlated with perceived stress, social support and self-efficacy. Besides, perceived stress, physical condition and working stress are 3 main influence factors of fatigue level. Hence more attention should be paid and some advisable measurements above could be taken to those emergency nurses with high perceived stress, poor sub-health state, heavy working pressure and low self-efficacy.

## Acknowledgments

We would like to express gratitude to the nurses of 6 hospitals for participating in this survey in spite of the busy work. We would like to say Thank you to the investigators for visiting hospitals repeatedly to collect the data, and the director of nursing department in Air Force Medical University, Lang Hongjuan, for actively contacting hospitals and strong support for this survey.

## Author contributions

WC contributed to the design of the study, the distribution of questionnaires, the collection of data, and the drafting and revising of the articles. GYL and ZXY contributed to the writing and polishing of article and the submission of contributions. XC contributed to the distribution of questionnaires, the collection of data. LHJ contributed to the design of the study, the distribution of questionnaires.
